# Interrater reliability in bipolar disorder research: current practices and suggestions for enhancing best practices

**DOI:** 10.1186/s40345-017-0111-7

**Published:** 2018-01-02

**Authors:** June Gruber, Lauren M. Weinstock

**Affiliations:** 1 Department of Psychology and Neuroscience, University of Colorado Boulder, 345 UCB Muenzinger D321C, Boulder, CO 80309-0345 USA; 2 Department of Psychiatry and Human Behavior, Brown University, 345 Blackstone Blvd, Providence, RI 02906 USA

## Background

Bipolar disorder (BD) is a complex and chronic psychiatric disorder associated with severe functioning difficulties. Typically marked by recurrent episodes of (hypo)mania and depression, and symptom overlap with several other psychiatric disorders (e.g., major depressive disorder, schizoaffective disorder, borderline personality disorder), accurate diagnosis presents a unique clinical challenge. Indeed, individuals with BD report an average of 9 years from initial presentation for treatment to an accurate diagnosis (Hirschfeld et al. 2003). At the same time, mounting evidence suggests that a substantial proportion of individuals previously diagnosed with BD may fail to meet formal diagnostic criteria upon structured interview, leading to false-positive cases in addition to the false-negative cases encountered in routine care (Zimmerman et al. 2008). Yet even when an accurate BD diagnosis is obtained, it remains difficult to correctly identify BD subtypes. Clinically, incorrect diagnosis may lead to delays in the delivery of appropriate, evidence-based care. From a research perspective, misclassification of individuals into diagnostic groups, for purposes of group comparison or for evaluation of novel treatment effects (e.g., Sachs et al. 2003), may bias or otherwise undermine the validity of research findings.

The challenges described above underscore the importance of accurate diagnosis and detection in BD. One approach to enhance diagnostic accuracy in BD research is through the establishment and reporting of interrater reliability (IRR). Surprisingly, there are no published guidelines describing this process. We discuss the importance of IRR, briefly note common features and variations, and suggest steps moving forward including greater transparency to facilitate replicability of practices.

## Current practices on interrater reliability in BD

IRR enables researchers to quantify the degree of agreement in ratings among two or more raters in clinical ratings (e.g., Ventura et al. 1998). IRR aids resolution of issues of differential diagnoses and overdiagnosis or underdiagnosis of BD (e.g., Hirschfeld et al. 2003; Zimmerman et al. 2008). As there are no published guidelines on IRR practices, we describe four common features.

First, IRR raters are trained in diagnostic criteria and clinical ratings, including listening to and coding of interviews from previous research participants, live observation, and supervised co-interviews. Additional training may include meeting an agreement criterion for clinical competency before conducting interviews (e.g., Weinstock et al. 2016).

Second, an investigator may choose to hold regular consensus meetings over the course of data collection. The goal of consensus meetings is to confirm the diagnosis (or score) is accurate or record a new corrected diagnosis (or score) established through discussion. Consensus meetings in clinical research are not designed to be a reliability tool; however, they may serve the function of maintaining rater consistency and preventing rater drift over time (e.g., Miklowitz et al. 2003). Raters may correct their scores when they come to the conclusion they have made an error or inaccuracy, although if disagreement remains and is an earnest difference of opinion, then it is kept as such given consensus meetings are not intended to minimize discrepancies based on honest differences of opinion (e.g., Sachs et al. 2003; Weinstock et al. 2016). Consensus meetings can occur weekly, monthly, or at important time anchors, or not at all when deemed unnecessary. Attendance includes some combination of supervisor(s), independent rater(s), original interviewer, and staff (e.g., Kosten and Rounsaville 1992). If a relevant member is unable to attend, notes are taken for consideration (e.g., Ong et al. 2017). All of these common variations fall within accepted standards of practice.

Third, each rater is assigned a subset of recorded interviews sampled randomly, quasi-randomly or nonrandomly to rate blindly and independently (i.e., prior to any group discussions). The proportion of blind ratings conducted may vary anywhere from < 10 to 100%, although a larger subset is preferable. Some may choose to skip this step due to the absence of subfield norms requiring it or by practical constraints such as staff shortages.

Fourth, current norms for reporting IRR to date are brief. Most studies include a description of the interviewer(s) and independent rater(s), proportion of interviews reviewed, and IRR statistics such as Kappa (for categorical diagnoses) or Intraclass Correlation Coefficients (for continuous measures). Often there is little to no mention of whether consensus meetings occurred and, if noted, minimal details are provided. It is often not specified whether the reported statistics reflect pre-consensus (i.e., how much did raters agree beforehand; Weinstock et al. 2016) or post-consensus (i.e., how much did raters agree after the meeting; e.g., Ong et al. 2017). Reported IRR values are commonly high (e.g., Skre et al. 1991) given the SCID “skip out” structure that reduces opportunities for disagreement (e.g., Joormann and Gotlib 2007). Although it is beyond the scope of this letter to provide a definitive conclusion for what constitutes acceptable IRR, we note that relevant commentaries have been provided elsewhere, suggesting variability in acceptable value ranges. For example, while some researchers consider kappas above 0.70 to indicate good agreement, others propose a lower goal of *k* = 0.40–0.60, but state that values as low as 0.20–0.40 are acceptable for psychiatric diagnoses (cf. Spitzer et al. 2012).

## Suggestions to enhance the best practices

In sum, IRR is utilized by researchers to facilitate diagnostic accuracy, which is especially challenging in BD research given its symptom complexity and challenges in differentiation from overlapping conditions. Surprisingly, there are no published guidelines discussing these common and accepted practices or what constitutes the best practice. We believe it is important to bring awareness to this issue and provide three concrete recommendations to motivate steps toward increasing transparency, avoiding confusion between and within research teams, and enhancing the best practices.

First, we recommend reporting IRR practices in greater detail which, up until now, have been reported by most researchers (ourselves included) in a fairly perfunctory manner. We recommend that researchers go beyond accepted practices to provide additional information including detailed descriptions of the consensus meeting process (and note if one did not take place), whether reported scores reflect pre- or post-consensus ratings, and results that correspond specifically to the data from participants included in the current analyses. These practices will greatly improve transparency in IRR reporting.

Second, increased transparency will open up the possibility of systematic and data-driven examination as to what actually constitutes the best practices. Such an examination might include systematic synthesis of the literature as well as quantitative meta-analyses examining which aspects of, or approaches to, IRR reliability best enhance and maintain diagnostic accuracy.

Third, it will be important to expand our scope beyond BD to gain insights into how other clinical literatures approach these practices. Given the transdiagnostic relevance of IRR, we can leverage important insights into the best practices from the literature (e.g., anxiety disorders) as part of a broader assessment of the best practices in clinical science and practice, while acknowledging unique issues for IRR in BD (e.g., overlapping diagnostic features with schizoaffective disorder).

Facilitating open conversation about common practices will stimulate discussion about the best practices in diagnostic decision making and promote greater transparency and cross-site replicability of BD studies. Our hope is that these critical self-examinations and set of recommendations will inspire other subfields to reflect and evaluate the status of reporting, conducting, and enhancing the best practices in IRR.
